# Sexual and Gender Minority Adolescents’ Preferences for HIV Pre-Exposure Prophylaxis Social Marketing Campaigns: Qualitative Preimplementation Study

**DOI:** 10.2196/60398

**Published:** 2025-01-17

**Authors:** Kathryn Macapagal, Juan Pablo Zapata, Junye Ma, Jacob D Gordon, Christopher Owens, Silvia Valadez-Tapia, Peter Cummings, Nathan Walter, Jim Pickett

**Affiliations:** 1 Institute for Sexual and Gender Minority Health and Wellbeing Northwestern University Chicago, IL United States; 2 Department of Medical Social Sciences Feinberg School of Medicine Northwestern University Chicago, IL United States; 3 Department of Psychiatry & Behavioral Sciences Feinberg School of Medicine Northwestern University Chicago, IL United States; 4 Joint Doctoral Program in Clinical Psychology San Diego State University/University of California San Diego San Diego, CA United States; 5 College of Nursing University of Cincinnati Cincinnati United States; 6 Department of Health Behavior School of Public Health Texas A&M University College Station, TX United States; 7 AIDS Foundation Chicago Chicago, IL United States; 8 Department of Communication Studies School of Communication Northwestern University Evanston, IL United States; 9 Jim Pickett Consulting Chicago, IL United States

**Keywords:** social marketing campaigns, sexual and gender minority, adolescent, HIV, pre-exposure prophylaxis, PrEP, human-centered design, implementation science, dissemination

## Abstract

**Background:**

Sexual and gender minority (SGM) adolescents in the United States are disproportionately affected by HIV. Pre-exposure prophylaxis (PrEP) is a highly effective biomedical HIV prevention method, but its awareness and uptake among SGM adolescents are low. There are no adolescent-centered PrEP social marketing campaigns in the United States that have the potential to increase awareness and interest in PrEP.

**Objective:**

To address this gap, this qualitative study aims to examine SGM adolescents’ needs and preferences regarding adolescent-centered PrEP social marketing campaigns.

**Methods:**

SGM adolescents from Chicago and its surrounding areas participated in web-based asynchronous focus groups from February to May 2021. Questions elicited their preferences for content, design, and delivery of SGM adolescent–centered PrEP campaigns. We used rapid qualitative data analysis and organized the findings around key components of social marketing, known as the 4 Ps: *product, price, place,* and *promotion.*

**Results:**

Participants (N=56) were aged 14 to 19 years (mean 18.16, SD 1.22 y), and 64% (36/56) of them identified as a racial or ethnic minority. Among the 56 participants, 70% (n=39) were aware of PrEP; however, 95% (n=53) did not know that PrEP could be prescribed to those aged under 18 years. Adolescents expressed a need for PrEP campaign messaging that provides simple, accurate, and easily accessible information (eg, what is PrEP, for whom PrEP is indicated, and where and how to access PrEP). For product and price, SGM adolescents wanted a campaign to address barriers to, costs of, and how to access PrEP and desired to know about other adolescents’ PrEP experiences to improve campaign relatability. For place and promotion, participants preferred digital campaigns on social media to reduce the possibility of embarrassment and stigma and increase the accessibility of health content.

**Conclusions:**

These findings lay the groundwork for designing adolescent-centered educational PrEP campaigns that prioritize both user preferences in PrEP marketing design and strategies to overcome common barriers to PrEP awareness.

## Introduction

### Background

Adolescents and young adults aged 13 to 24 years in the United States are disproportionately affected by HIV, accounting for 19.3% of new diagnoses in 2022 [1]. In 2019, of the 25,552 new HIV infections among sexual minority men in the United States, 6386 (25%) were aged 13 to 24 years [2]. Surveillance data in Cook County, Illinois, an Ending the HIV Epidemic priority area that includes Chicago and its surrounding areas, show that 82.3% of HIV cases among youths aged 13 to 29 years were among men who have sex with men, 59.7% of the cases were among Black sexual and gender minority (SGM) youths, and 20.3% of the cases were among Latino SGM youths [3].

Pre-exposure prophylaxis (PrEP) refers to HIV prevention medication regimens that currently include oral emtricitabine-tenofovir disoproxil fumarate (brand name Truvada) or emtricitabine-tenofovir alafenamide (brand name Descovy) and long-acting injectable cabotegravir (brand name Apretude). PrEP is a highly effective biomedical prevention strategy, preventing up to 99% of HIV infections when taken as prescribed [4,5,6]. Despite the US Food and Drug Administration’s approval of oral PrEP for individuals aged under 18 years in 2018 and long-acting injectable PrEP in 2021 [7], PrEP uptake remains subpar in this age group [8,9]. One study that used US pharmacy claims data to estimate adolescent PrEP use [10] found that of the 6444 youths aged 13 to 19 years who were prescribed PrEP in 2021, just 12% were aged under 18 years, and most were adolescent girls. Across several studies using convenience samples, self-reported PrEP uptake among SGM youth aged 14 to 17 years has been consistently low, ranging from 2% to 5.9% [8,11-14].

Awareness and knowledge of PrEP are 2 significant barriers to its use among adolescents. In studies based in the United States, PrEP awareness among SGM adolescent samples has varied from 15% to 55% [12-15], with higher rates observed in studies consisting of participants who had previously taken part in HIV prevention research where they learned about PrEP. Several demographic, social, cultural, and structural factors relate to SGM youth PrEP awareness, including older age, greater educational attainment, and better access to sexual health care [16-20]. Research also suggests that adolescents have limited knowledge about PrEP beyond the fact that it prevents HIV infection and assume that PrEP is only available for cisgender men or those aged ≥18 years [11,12]. When adolescents are aware of PrEP, they report moderate to high levels of willingness to take it; for example, 2 survey studies showed that 64.9% [9] to 78.5% [8] of adolescent sexual minority men aged 13 to 18 years were willing to use PrEP. Furthermore, even when SGM adolescents are aware of PrEP, their willingness to use it may be moderated by a variety of developmental, socioeconomic, and sociocultural barriers to obtaining and adhering to PrEP. These include concerns about parental involvement, affordability, the need for regular clinic visits but the difficulty in accessing them independently, side effect concerns, and a lack of health care provider training in adolescent PrEP provision [8,10,12,17,21-25].

Social marketing campaigns, which integrate marketing, psychology, and public health principles, have immense potential to promote PrEP awareness and uptake among SGM adolescents, creating positive changes in attitudes, behaviors, and awareness for the greater good of society [26,27]. The key components of social marketing are often referred to as the 4 Ps [28,29]. *Product* includes messages about the desired behavior and its attributes, its health benefits (eg, PrEP awareness, uptake, and HIV prevention), and perceived nonhealth benefits of the behavior (eg, fulfilling social needs). *Price* refers to real and perceived financial or practical costs or other barriers to engaging in the desired behavior from the consumer’s perspective and strategies to lower these costs. *Place* involves determining where the focal audience engages in the behavior (in this case, where one obtains PrEP) and implementing ways to make the desired behavior, service, or product more accessible. *Promotion* entails the ways persuasive messages are designed and delivered to the desired audience [28]. Other models also include a fifth P, *policy,* which includes policy-level barriers and facilitators to engaging in the desired behavior that should be communicated to the audience [29]. For instance, in some US states, PrEP is explicitly included in laws permitting adolescents to access certain types of health care without parental consent (eg, mental health and substance use treatment; prevention, testing, and treatment of sexually transmitted infections [STI] and HIV; and other sexual and reproductive health care) [30].

Social marketing campaigns take effect on both population and individual levels [31]. In the case of adolescents, a PrEP social marketing campaign could reach them quickly and directly through multiple forms of media (eg, social media, websites, and posters) that circumvent the need to rely on parents, health care providers, and educators who may or may not deliver or even know about this information. One example of such a campaign is PrEP4Love [32,33], a Chicago-based PrEP awareness initiative designed for Black adult gay and bisexual men and transgender and cisgender women. PrEP4Love reached 41 million unique viewers and was associated with a 3-fold increase in calls to a PrEP hotline over the 2 months following the campaign launch, most of whom were interested in learning more about or being linked to PrEP [32,33]. The success of the campaign in Chicago led to its adoption by a French AIDS service organization to raise PrEP awareness across France [34].

Although adolescent-inclusive sexual health campaigns exist, such as Get Yourself Tested [35] and National Youth HIV/AIDS Awareness Day [36], few specifically include information about or an emphasis on PrEP. Social marketing draws from exchange theory, which suggests that individuals consider perceived costs and benefits when making decisions and focuses on segmenting the population into distinct audiences (ie, market segmentation) that are more likely to respond to a particular campaign or campaign component [37,38]. As such, social marketing campaigns can be designed to be developmentally appropriate for adolescents to highlight their specific preferences and needs. For example, the content and imagery of PrEP4Love [32,33] were clearly sensual and sexual in nature, which may make many adolescents uncomfortable; approaches that elicit different emotions, such as empowerment or affirmation, could be more resonant with younger audiences [39,40]. Moreover, where campaigns are placed is critical in ensuring that the campaign reaches its intended audience. For instance, PrEP4Love was often promoted in person (eg, in health clinics, public transit, and nightlife establishments) with a website, hotline, and social media presence. However, SGM adolescents may not be able to access the same physical or web-based places (eg, bars and adult-focused sexual health clinics) or may not pay attention to campaign materials in public spaces that portray intimacy among adults.

### This Study

In this study, we used a “designing for dissemination and sustainability” approach, which uses methods and principles from dissemination and implementation science, communications, marketing, community engagement, and human-centered design. This interdisciplinary approach enables the tangible products of public health research to better meet the long-term needs of the intended audience and settings [41]. Following this approach, we directly engaged SGM adolescents in preparatory implementation research via web-based focus groups to inform whether and how to undertake an SGM adolescent–centered PrEP social marketing campaign in the Chicago area. Focus group questions sought to understand their perspectives on existing PrEP and sexual health campaigns and their preferences for campaign content, design, and implementation. At the end of the focus groups, we invited SGM adolescents to develop initial prototypes of digital marketing materials that they thought may resonate with a teenaged audience [42,43].

## Methods

These data were collected in the context of a larger study that focused on lesbian, gay, bisexual, transgender, and queer (LGBTQ+) adolescents and supportive adults’ perspectives on the need for an adolescent-centered PrEP campaign in the Chicago area and what each group desired out of such a campaign. Findings from interviews with adult participants are reported elsewhere [24].

### Recruitment

SGM adolescents in Cook County, Illinois, were recruited from February to May 2021 via social media advertisements (eg, Instagram [Meta Platforms] and Snapchat [Snap Inc]); the Illinois PrEP Working Group listserv; registries of individuals who had expressed interest in research study participation through Northwestern University’s Institute for Sexual and Gender Minority Health and Wellbeing and the Third Coast Center for AIDS Research; and through the research team’s partnership with AIDS Foundation Chicago, a local HIV advocacy and service organization. Social media advertisements were designed to reach adolescents interested in SGM culture (eg, LGBTQ+ organizations and important figures), and study staff reached out to individuals in the registries via SMS text message or email to inform them of a new study opportunity. Social media advertisements and registry outreach directed prospective participants to the study’s web-based screener. Eligible adolescents included those who (1) were aged 13 to 19 years; (2) lived in Cook County, Illinois; (3) identified as a sexual or gender minority individual; (4) self-reported HIV negative or unknown status; (5) had access to the internet; and (6) could read in English at an eighth-grade level. We used purposive sampling to enroll up to 20% transgender and nonbinary youths and at least 80% cisgender sexual minority youths to ensure representation of youths across the LGBTQ+ spectrum and aimed for a sample composition of at least 60% racial and ethnic minority youths to reflect the demographics of the Chicago area.

### Ethical Considerations

All study procedures were approved by the Northwestern University Institutional Review Board (STU00213036). A waiver of parental permission was obtained for minor adolescents, as the study was determined to pose minimal risk. Following completion of the web-based screener, those who were preliminarily eligible were given a web-based consent form and responded to 4 multiple-choice items to assess their comprehension of the study procedures; those who failed these items twice after being given the opportunity to reread the consent form were deemed ineligible. Then, study staff contacted preliminarily eligible adolescents to participate in a Zoom (Zoom Communications, Inc) call, during which staff confirmed that the respondent was indeed an adolescent, met the eligibility criteria, understood the study procedures, and agreed to a code of conduct for focus group behavior (eg, no harassment or bullying, respectful language, and keeping discussion confidential). To protect their privacy, participants were encouraged to select a username for the focus groups that they did not use elsewhere on the Internet and were not permitted to include identifying information in their usernames. Throughout the study, participants were reminded that they could contact the study team at any time via study SMS text message or email with questions or concerns. At the end of the study, information about study payment (US $40 Amazon gift card sent via email) and HIV and PrEP resources were provided. Data presented here are deidentified for privacy.

### Procedures

After enrolling, participants were sent a link to a 25-minute web-based baseline survey via email or text. Participants could select “I don’t want to answer” for any item in the survey. Study staff then sent adolescents instructions to access an asynchronous focus group website developed using vBulletin (Internet Brands), web-based forum software that the study team had used in previous research [11,12]. Web-based focus groups have long been used in social science research due to their accessibility and flexibility (eg, they can be conducted synchronously or asynchronously), including in the research team’s own work [44,45]. They have also been shown to provide data quality comparable to that of in-person focus groups [46]. Participants accessed the focus group website with unique log-ins. There, they could view links to an introductory forum and 7 forums focused on different study topics, with 1 to 5 questions within each topic (refer to Focus Group Guide section). The introductory forum included a video introducing the study team to participants, an overview of ground rules, and an icebreaker. Participants contributed by typing their responses or uploading content at their convenience. The focus group website was available for 7 days, and participants were strongly encouraged to complete all questions within the first 2 to 3 days to ensure sufficient time for the moderators to ask follow-up questions and for participants to interact with each other’s written responses. To increase the likelihood of participant responses, probes used the @username function to trigger notifications. In addition, daily text messages or email reminders that included a direct link to questions and probes participants had not yet responded to were used to increase their engagement. We conducted 5 focus groups, with each group including 11 to 17 participants. Each group was facilitated by 2 moderators who were trained in qualitative methods. Adolescents who answered all questions on ≥4 of the 7 topics were considered completers and eligible for payment.

### Focus Group Guide

The focus group guide assessed participants’ reactions to existing PrEP campaigns and preferences for future adolescent PrEP campaigns. Questions were adapted from an unpublished needs assessment previously conducted by local community leaders working in HIV prevention and care, including one of the coauthors (JP), to inform the development of adult-focused English and Spanish PrEP social marketing campaigns in Chicago. Additional questions were added by the study team to be inclusive of adolescent experiences based on findings from empirical research on adolescent barriers and facilitators to PrEP and adolescents’ preferences for receiving information about sexual health [11,13,25]. Textbox 1 provides examples of focus group questions and topics.

First, we presented existing PrEP and HIV campaigns (PrEP4Love [32-34], Get Yourself Tested [35], and the National Youth HIV/AIDS Awareness Day Campaign [36]) to give participants a reference point for public health campaigns focused on HIV and sexual health and those focused on youths versus adults, given that no PrEP campaigns for adolescents existed at the time. Questions assessed participants’ reactions to these campaigns, areas of improvement, and comparisons. Next, we assessed adolescents’ preferences for message delivery and dissemination, preferences on campaign design and content, developmental and cultural considerations, and perspectives on messaging for parents and injectable PrEP (not described in this study). Finally, a creative activity prompted adolescents to design mock PrEP campaign content; provide a sample image or video of a campaign they liked and an explanation; or develop a mood board reflecting colors, feelings, or content that might inspire a future campaign. At the end of the group, participants were invited to share their feedback, ask questions, or share additional thoughts they may have had that were not covered. Each focus group transcript was saved as a deidentified Microsoft Word document on password-protected servers. Upon downloading focus group transcripts, study staff disabled further user comments and visibility of the group to all users.

**Focus group topics and sample questions.**

**Topic 1: Your thoughts on the PrEP4Love campaign**

- What works well in this campaign? What doesn’t work so well in this campaign?
- PrEP4Love was designed for adults. What changes would you make to the campaign to make it more appealing and interesting to LGBTQ+ teens?

**Topic 2: Your thoughts on the Get Yourself Tested and National Youth HIV/AIDS Awareness Day campaigns**

- Compare and contrast the PrEP4Love, GYT, and NYHAAD campaigns. What aspects of each campaign do you like the best? What do you not like?
- Which of the aspects you mentioned above are the most important to you to include in a PrEP campaign for LGBTQ+ teens?
- What, if anything, might be missing from any of these campaigns that would be important to include in a PrEP campaign for LGBTQ+ teens?

**Topic 3: Where should PrEP campaigns go?**

- What in-person spaces would be good places to post ads/share info about PrEP for LGBTQ teens?
- What online spaces/platforms would be good places to post ads/share info about PrEP for LGBTQ teens?
- If you were to design a PrEP video for TikTok, what would your video look like?
- If you were to design a PrEP post or story on Instagram, what would it look like?

**Topic 4: What should PrEP messages for LGBTQ+ teens in Chicago look like**

- What words and/or phrases would capture your and other LGBTQ+ teens’ attention?
- What types of images would you want to include in your PrEP campaign?
- If you wanted to show people in your PrEP campaign, what type of people would you show?

**Topic 5: LGBTQ+ teens’ feelings and reactions to PrEP campaigns**

- How would you want a new PrEP campaign to make you and other LGBTQ+ teens feel?
- How (if at all) should teen PrEP campaigns differ for younger versus older LGBTQ+ teens? LGBTQ+ teens who ever had sex versus those who never had sex? LGBTQ+ teens who are out versus those who are not out? Teens who identify as LGBTQ+ versus teens who are questioning or do not identify as LGBTQ+ but have sex with same sex/gender partners?

**Topic 6: PrEP messaging for adults and other forms of PrEP**

- How do you think PrEP messages for parents and other adults need to be DIFFERENT from those aimed at LGBTQ teens? How should they be SIMILAR?
- In your opinion, what information is most important in an ad about teens and PrEP designed for parents/guardians?
- If you were designing a campaign for injectable PrEP, what is the one piece of information you’d want in it? How would it be different from a PrEP campaign focused on pills?

**Topic 7: Your examples of LGBTQ+ teen PrEP campaigns!**

- Option 1: Find an example of health-related (or PrEP/HIV/sexual health-related) content that you LIKE or LOVE! Then, explain what you like about it, and how it relates to your ideas for what a PrEP campaign for teens should be like.
- Option 2: The DIY route. Here are some ideas to get you started, but if you have other ideas feel free to run with it! Use apps like Instagram or Snapchat to edit/draw on/type on the campaigns we shared with you, create a mood board/collage to share the vibe and look you’d want your campaign to have, draw something on your phone.

### Measures

#### Sociodemographic Characteristics

Participants self-reported their age, race, ethnicity, sex assigned at birth, sexual orientation identity, gender identity, outness to parents or guardians, parents’ or guardians’ acceptance of SGM identity, and sources of sexual health information. Additional investigator-created items assessed participants’ HIV and STI testing history, previous sexual experience, and engagement in HIV transmission risk behavior, and other items assessed perceived HIV risk [47].

#### General PrEP Questions

Investigator-created items assessed perceived norms of PrEP uptake (“I’d like you to imagine 100 adolescents like you. How many of these 100 adolescents do you think are likely to use PrEP”), PrEP awareness before the study (*yes* or *no*), and lifetime uptake (*yes*; *no*). Participants also self-reported perceived difficulty (1=*not at all difficult* to 5=*very difficult*) and confidence in their ability to access PrEP (1=*not at all confident* to 5=*very confident*).

#### PrEP Attitudes and Stigma

A scale developed by Walsh [48] in 2019 was used to assess participants’ attitudes and perceived stigma toward PrEP. A total of 5 items evaluated perceived effectiveness, safety, and adherence to PrEP, while another 5 items addressed stigma concerns associated with PrEP (eg, “People who take PrEP are promiscuous”). Responses were on a 5-point Likert scale (1=*strongly disagree* to 5=*strongly agree*). PrEP attitude scores ranged from 5 to 25, with higher scores indicating a positive view of PrEP. Stigma scores also ranged from 5 to 25, with higher scores indicating greater perceived stigma.

### Data Analysis

Descriptive analyses for all quantitative variables were conducted in R software (x64, version 3.6.1; R Foundation for Statistical Computing). Focus group data were analyzed deductively using rapid qualitative analysis [49], a method commonly used in public health and implementation research to quickly identify actionable themes in qualitative data. The results of such analyses have been shown to correlate highly with traditional qualitative analysis [50,51]. First, study staff exported participants’ responses from the Microsoft Word group-level transcripts to tables in Microsoft Excel, with different tabs created for each focus group topic, and responses were cross-validated for accuracy. Two coders reduced data by emphasizing informative sections of participants’ responses and removing redundant or filler words. This process resulted in concise data that spoke to the study objectives. Following data reduction, the coders independently examined all excerpts, assigned codes to all excerpts, refined the code definitions iteratively, and held discussions to reconcile differences. Data were then organized conceptually and reported according to the 4 Ps model. Quotes presented in the Results section include participants’ age, gender, race, ethnicity, and sexual orientation.

## Results

### Screening, Enrollment, and Analytic Sample Characteristics

Overall, 348 individuals accessed the eligibility screener, of whom 126 (36.2%) completed the screener. Of the 126 participants who completed the screener, 96 (76.2%) were preliminarily eligible. Of the preliminarily eligible individuals, 68% (65/96) were enrolled after the Zoom call. Then, 94% (61/65) completed the baseline survey and 92% (56/61) answered ≥1 question in the focus group, and 89% (50/56) completed the focus group (ie, responded to all questions in the majority; 4/7, 57% of the topics); 94% (47/50) participants responded to all questions in all 7 topics. The characteristics of the analytic sample are in Table 1.

**Table 1:** Participant demographics (N=56).

Variables		Values
Age (y), mean (SD)		18.16 (1.22)
**Age (y), n (%)**		
	14	1 (2)
	15	2 (4)
	16	5 (9)
	17	16 (29)
	18	16 (29)
	19	16 (29)
**Sex assigned at birth, n (%)**		
	Male	28 (50)
	Female	28 (50)
**Race and ethnicity, n (%)**		
	Asian	6 (11)
	Black	9 (16)
	Hispanic	12 (21)
	Multiracial	9 (16)
	Non-Hispanic White	19 (34)
	No answer	1 (2)
**Gender identity, n (%)**		
	Agender	1 (2)
	Cisgender man	20 (36)
	Cisgender woman	12 (21)
	Transgender man	5 (9)
	Transgender woman	3 (5)
	Genderqueer	4 (7)
	Gender nonconforming	3 (5)
	Questioning	6 (11)
	No answer	2 (4)
**Sexual orientation identity, n (%)**		
	Asexual	1 (2)
	Bisexual or pansexual	23 (41)
	Gay	20 (36)
	Lesbian	3 (5)
	Queer	6 (11)
	Questioning	3 (5)
**Prior sexual experience (allowed multiple options), n (%)**		
	People with penises	33 (59)
	People with vaginas	17 (30)
	Never had penetrative sex	19 (34)

Participants could indicate multiple sources from where they heard of the study. Most participants heard of the study through paid social media advertisements (23/56, 41% via Instagram; 10/56, 18% via Facebook; and 6/56, 11% via Snapchat). Other sources included a registry email or text message (7/56, 13%), an LGBTQ+ organization (5/56, 9%), a friend (5/56, 9%), or a clinic or hospital (2/56, 4%), and 2 (4%) participants indicated that they heard about the study elsewhere (a family member and an email newsletter for a different study for LGBTQ youth). Participants said that their main sources of sexual health information were social media (30/56, 54%), LGBTQ+ websites (15/56, 27%), and porn websites (14/56, 25%). More than half (37/56, 66%) of the participants had prior sexual experience, 48% (27/56) reported condomless penetrative sex, and 13% (7/56) had sex with someone who tested positive for HIV or who was of unknown status. Less than half self-reported that they had previously tested for HIV (24/56, 43%) or STIs (18/56, 32%).

One-third (19/56, 34%) of the participants worried about testing positive for HIV, and 24% (13/56) perceived themselves at risk for HIV infection. ANOVAs revealed significant group differences in perceived risk for HIV infection by gender identity (*F*_7,55_=2.47; *P*=.03); Bonferroni-corrected pairwise comparisons showed that those who identified as men perceived greater risk than those who identified as women (mean 2.56, SD 1.26 vs mean 1.4, SD 0.63, respectively). There were also significant group differences in sexual orientation (*F*_5,55_=2.79; *P*=.03) whereby participants identifying as gay perceived greater risk than those identifying as bisexual or pansexual (mean 2.80, SD 1.20 vs mean 1.78, SD 0.90, respectively). There were no significant differences in perceived HIV risk by age, race, ethnicity, sex assigned at birth, or having ever been tested for HIV or STIs.

Most (39/56, 70%) participants were aware of PrEP. Only 4% (2/56) had taken it, and 95% (53/56) were unaware that it could be prescribed to those aged <18 years. In addition, 38% (21/56) of the participants found accessing PrEP easy, while 50% (28/56) were confident in their ability to do so. Adolescents viewed PrEP positively regarding effectiveness, safety, and adherence (mean 19.93, SD 2.91; range 5-25) and had moderate stigma levels (mean 11.91, SD 4.39; range 5-25). More details are provided in Table 2.

**Table 2:** Pre-exposure prophylaxis (PrEP) awareness, use, and attitudes (N=56).

Variables		Values
Perceived norms (“Out of 100 adolescents like you how many do you think are likely to use PrEP?”), mean (SD)		37.88 (24.11)
PrEP attitudes, mean (SD; range)^a^		19.93 (2.91; 5-25)
PrEP stigma, mean (SD; range)^b^		11.91 (4.39; 5-25)
**Participants’ awareness of PrEP before participating in the study, n (%)**		
	Yes	39 (70)
	No	16 (29)
	No answer	1 (2)
**Do you know any teens aged under age 18 who are taking PrEP? n (%)**		
	Yes	3 (5)
	No	53 (95)
**Lifetime PrEP use, n (%)**		
	Yes	2 (4)
	No	54 (96)
**Perceived difficulty in accessing PrEP, n (%)**		
	Very easy	7 (12)
	Somewhat easy	14 (25)
	Neither difficult nor easy	17 (30)
	Somewhat difficult	17 (30)
	Very difficult	1 (2)
**Confidence in the ability to access PrEP, n (%)**		
	Not at all confident	4 (7)
	Somewhat unconfident	9 (16)
	Neither confident nor unconfident	15 (27)
	Somewhat confident	22 (39)
	Very confident	6 (11)

^a^Higher scores indicate more positive attitudes toward PrEP.

^b^Higher scores indicate greater stigma against PrEP use.

### Reactions to Existing PrEP Campaigns

Participants were presented with the PrEP4Love, Get Yourself Tested, and the National Youth HIV/AIDS Awareness Day Campaigns and were asked to react to the design, images, and written messages of each campaign. Adolescents generally favored campaigns that highlighted “diversity” in sexual and gender identities, body type, and couple types (eg, couples depicting transgender and cisgender individuals) and disliked campaigns that lacked representation of these groups, including adolescents:

I really like that the images show many different couples, regardless of race, gender, sexuality, etc.

Asian gay man aged 19 years

That said, they noted that the intentionally sensual nature of the PrEP4Love campaign was off-putting:

It seems too sexual/sensual and likely would make me look away before reading the [tag]line.

Non-Hispanic White bisexual or pansexual man aged 17 years

Regarding campaign taglines and copy, SGM adolescents favored “straightforward” messages, particularly those that challenged HIV stigma:

The simplicity of the campaign definitely works well. I think that it conveys simple information...while simultaneously being informative enough.

Hispanic bisexual man aged 17 years

Respondents expressed dislike for messages that were too broad, lacked clarity, or failed to provide PrEP-related resources or information. Adolescents noted that the National Youth HIV/AIDS Awareness Day campaign messages were “too busy and wordy” (non-Hispanic White bisexual or pansexual woman aged 17 years), and that the Get Yourself Tested campaign had a clear call to action but lacked further resources:

For GYT...I do think that there could have been a little bit more information.

Hispanic questioning woman aged 19 years

Participants preferred existing campaigns with “bright, eye-catching designs” and disliked campaigns with fonts that were difficult to read or those that did not feature teenaged models.

### Preferences for Future Adolescent-Centered PrEP Campaigns

In this section, participants’ preferences regarding the design of future adolescent-centered PrEP campaigns were organized based on the 4 Ps framework [29].

#### Product

Participants’ comments suggested that social marketing campaigns should specify certain details about PrEP and HIV prevention for adolescents; mention its benefits among youths who have used PrEP; include messages that normalize the health, experiences, and relationships of SGM adolescents; and provide ample resources about PrEP, including information about how to talk to a health care provider about PrEP.

Participants described wanting campaigns to give information regarding the purpose and effectiveness of PrEP and how to access it:

I think it should include more...about HIV, what PrEP is, and the cost of PrEP.

White queer, genderqueer adolescent assigned male at birth aged 19 years

Participants also desired more tangible information about PrEP statistics and adolescents’ lived experiences obtaining and being on PrEP:

Including statistics, quotes, and real experiences from adolescents would make it more relatable and emphasize the importance.

Hispanic White questioning woman aged 19 years

Other participants also emphasized the importance of including resources about PrEP access, such as offering a comprehensive list of local adolescent-friendly PrEP providers, information about talking to a provider about PrEP, and resources about insurance coverage and paying for PrEP. A , mentioned as follows:

I think you’re missing financial information [is it free?], contact information...and you can say “talk to your doctor” all you want but like, when I was [younger], I didn’t have a doctor. Who would I go to?

Black transgender man questioning their sexual orientation aged 19 years

A participant summarized common sentiments among the group regarding product information and how to frame this information to appeal to the audience:

I think that the dosing, how to get it, why it’s good to take, and information regarding how it works [in a simplified manner], are all good things to include. If you just have a poster saying, “Take PrEP,” nobody will take it, but if you say something closer to “Take PrEP, a once daily medication that keeps you 99% safe from HIV,” it will be more effective.

White bisexual woman aged 19 years

Respondents noted that PrEP messaging centered on the needs of SGM adolescents, and not exclusively focusing on SGM populations, would likely be beneficial to adolescents regardless of background or identity. This was not only perceived to help normalize the health, experiences, and relationships of SGM adolescents but also showed cisgender heterosexual adolescents that PrEP could be for them as well (eg, campaign materials that say “PrEP is for everyone” and depict adolescents of various sexual orientations and genders). This focus on inclusive yet relatable messaging is reflected as follows:

The way that my friends explain PrEP to me is...you take the pill once a day, kinda like birth control [which is reassuring because we all know someone on birth control].

Black gay man aged 18 years

Furthermore, youths described how portraying SGM adolescents authentically, “just being themselves, like watching TV or enjoying outdoor activities” (Black gay man aged 17 years), can facilitate this sentiment in PrEP campaigns. Finally, another benefit of adolescent-centered PrEP campaigns is that they can foster a sense of inclusion and empowerment among adolescents, which can help motivate them to consider taking action to use PrEP:

I want to feel valued, supported, and seen, where people genuinely care about my sexual health and well-being.

Asian gay man aged 18 years

#### Price

Most comments identified in this domain referenced the price of PrEP (eg, “A big part would be price of PrEP...and any side effects,” as mentioned by a Hispanic gay man aged 16 years), the effort involved in accessing it, and the possibility of stigma and embarrassment with engaging with the campaign or seeking PrEP. Fewer adolescents expressed concerns about side effects or the medication regimen itself.

One transgender adolescent explained the potential concerns regarding PrEP that a campaign may help address by relating PrEP to their experiences seeking gender-affirming care:

Like when I was younger, I really wanted to go on hormones but...it felt like it would be impossible to get because I didn’t fully understand how to access it and a lot of people were telling me how complicated it was. So, I think teens need reassurance that it is simple/easy to obtain prep and maybe even include a step-by-step guide on how to go on it?

White bisexual transgender man aged 19 years

Other participants described how engaging with a PrEP campaign or talking about PrEP to a provider or clinic staff member could be embarrassing or elicit judgment from others, which campaigns should proactively address:

I don’t want to feel ashamed of talking about the topic. I want to feel respected and that I don’t need to fear that others might judge me.

Black gay woman aged 18 years

Similarly, other youths reported parental involvement as a potential barrier to accessing PrEP:

If we go to our doctors for a routine checkup, we’d be scared to talk about sex in general...because most teens are going to be intruded on by parents...it’s surely a roadblock for many teens.

Black gay man aged 18 years

Moreover, another participant described ensuring that the campaign had multiple ways to speak with a person about PrEP, such as a text line or web-based chat function, given young people’s reluctance to speak on the phone:

I would include specific ways to get into contact about getting PrEP besides just a phone call because many teens would be way too anxious to call a stranger about that.

Non-Hispanic White gay man aged 19 years

Participants were asked to articulate possible drawbacks of adolescent PrEP campaigns focused on specific populations (eg, SGM individuals, racial and ethnic minority individuals, and younger adolescents). Although campaigns specifically for SGM individuals can ensure that PrEP awareness is increased among this population as well as address certain concerns that these populations may have in relation to PrEP (“Black and Latinx people lack medical access and are wary of the medical system because it has mistreated them lots of times,” as mentioned by a Hispanic bisexual woman aged 18), participants described that this approach could downplay HIV susceptibility among cisgender heterosexual adolescents and perpetuate stigma within and toward the SGM community. A Black gay woman aged 18 years summarized this concern, stating the following:

...it may convey the message that HIV solely affects the LGBTQ community, rather than being a universal virus that impacts all of us.

Others suggested how campaigns should be attuned to developmental differences in adolescents, with older adolescents being more comfortable with more explicit sexual information or references and younger adolescents possibly experiencing stigma in searching for sexual health information:

I feel like some younger people may feel embarrassment or shame in looking into preventative measures.

Non-Hispanic White bisexual woman aged 19 years

#### Place

For the domain of place, participants primarily provided recommendations on how specific individual “champions” or locations that can support youth well-being and health behavior could effectively communicate where and how adolescents could access PrEP, particularly for those who may be less familiar with navigating sexual health care. The following participant quoted below and others described how key influential adults and organizations (eg, schools and accepting churches) could play a role in supporting adolescents’ access to PrEP, even if these locations did not offer PrEP themselves:

...every school should have guidance counselors or at least a support group for LGBTQ+ teens. I go to a catholic school and recently this year we made a new group called “Unidos” [‘Together’ in English]. That group helped me so much with resources and it made me feel represented.

Hispanic gay man aged 15 years

In addition, physical spaces recommended by participants included schools and public and entertainment venues, such as malls, public libraries, public transportation, and restaurants. These locations were favored due to their accessibility and convenience for SGM adolescents. For example, some youths identified public libraries as places that are accessible to and safe for adolescents, where they could learn more about PrEP with relative privacy:

I also think that libraries could work well because they are very trustworthy and when teenagers go to libraries, they are often alone to study or find books.

White bisexual woman aged 17 years

Participants also recommended advertising in LGBTQ+ serving organizations, clinics such as Planned Parenthood, pharmacies, and health care provider waiting rooms. These participants indicated that the placement of marketing materials in waiting rooms could be beneficial:

Family doc waiting rooms...if you have to talk to your doctor about PrEP a reminder before going in would be good.

White bisexual woman aged 18 years

The placement of marketing materials in pharmacies and hospitals was echoed by other participants as follows:

...because it will generate a lot of trust from teens because it is in a healthcare facility. When you go get a check-up it will be easy to remember, and teens might even like to bring it up with their doctor in private.

Black gay woman aged 18 years

#### Promotion

Participants’ feedback predominantly focused on the element of promotion, and they articulated strategies that can raise awareness about PrEP among SGM adolescents through carefully crafted content, appealing graphic design, and strategic placement. Finally, participants were also asked to give examples of what these campaign materials might look like.

### Campaign Content

This element was distinct from “product” in that it focuses more on message framing, tone, word choice, and market segmentation than the features or benefits of the product itself. Regarding the phrasing of adolescent PrEP messages, participants expressed a preference for concise messages presented in casual language to increase engagement. Furthermore, adolescents highlighted that a campaign should have an affirming and empowering tone that is community-oriented, with several adolescents offering phrases such as “your health matters” or “let’s stop HIV together.”

Considering the influence of peers and role models on adolescents, participants also suggested including celebrities and social media influencers in future PrEP campaigns to increase campaign appeal:

I would feature a well-known celebrity who is attractive and has been a strong supporter of the LGBTQ community.

Hispanic gay man aged 16 years

Participants frequently recommended involving adult authority figures, such as scientists, health care providers, and educators, for their credibility and expertise in addressing concerns about PrEP side effects or potential long-term effects, provided their delivery was empowering and supportive rather than patronizing:

...doctors/healthcare providers. This would help make PrEP campaigns feel more reliable and trustworthy.

Black bisexual or pansexual woman aged 17 years

Moreover, SGM adolescents suggested featuring parents and other adults who may play influential roles in adolescents’ health care (eg, “parents who feel more comfortable with their adolescents taking PrEP,” as mentioned by an Asian gay man).

Although participants felt that adolescents should get the same access to basic PrEP information regardless of where they lived or who they were, they acknowledged the importance of content segmented by population to address key differences:

As someone who went to school in the ‘burbs but lives in the city, it tends to be a bit more conservative up there, so I would just be aware of that.

White queer man aged 19 years

you’d have to advertise in different types of locations, as suburban teens don’t take public transportation for example.

Hispanic bisexual man aged 17 years

This included creating Spanish language materials to reach the large Spanish-speaking community in Chicago, which could ultimately improve access:

...this community, which I’m also a part of, could benefit [by] having ads that are also in Spanish. If possible, just adapting ads to be more approachable and informative for other languages other than English.

Hispanic bisexual woman aged 18 years

### Campaign Design

Regarding campaign design, adolescents preferred eye-catching graphics, including cartoons, avatars, and infographics:

...well thought out color schemes and easy-to-read fonts, along with statistical evidence that explains the effectiveness of PrEP [such as pie charts or bar graphs].

White gay man aged 18 years

Adolescents suggested that campaigns should avoid overly sexual images for discretion, with a Hispanic bisexual or pansexual woman aged 18 years stating, “staying away from anything that reinforces stereotypes or is overly sexual (due to parents).” Moreover, participants were clear that the campaign should avoid seeming too slick or corporate, which was viewed as untrustworthy, as described by a participant:

...you need to get that attention in a way that is personable, to show that this isn’t just something being peddled by a brand, and that it is something genuine and important, from queer folk, to queer folk...Make it real, make it raw, adding polish at the end removes a level of authenticity that is necessary for this level of trust.

White queer transgender woman aged 19 years

Although adolescents acknowledged that there are benefits to market segmentation, they were emphatic that the campaign design should also be inclusive and diverse as they believed everyone could benefit from learning about PrEP:

I wouldn’t necessarily exclude white bodies, but I would put an emphasis on people of color...it hurts literally no one to include them more and really only benefits. also, I think there shouldn’t be much distinction between cis and trans [people regarding campaign content], but I do think that we need a bit more trans representation.

Non-Hispanic White queer man aged 19 years

### Campaign Placement

Participants emphasized that an adolescent-centered PrEP campaign should predominantly be digital to reach this age group regardless of their location. They suggested using smartphone apps and LGBTQ+ websites; social media, such as Instagram, TikTok, and Tumblr; dating apps (eg, Grindr and Tinder); music streaming apps (eg, Spotify); and mobile games. Among these, social media, particularly TikTok, emerged as the most widely discussed digital platform for campaign dissemination as it was perceived to have “a significant reach and tends to be LGBTQ+ friendly” (Hispanic questioning woman aged 19 years). Participants suggested avoiding Facebook (“I don’t know many adolescents who actually use it. It’s more popular with adults and the older generation,” as mentioned by a Hispanic bisexual or pansexual woman aged 18 years) and digital spaces that feature skippable advertisements (eg, “ads on video streaming services because many of us skip them and don’t even pay attention to them,” as mentioned by a Hispanic questioning man aged 16 years) or have a reputation for tolerating more toxic Internet discourse (eg, Reddit and Twitter (subsequently rebranded X).

Regardless of medium, participants suggested that physical and digital campaign assets all lead to centralized resources:

Having a QR Code that leads to a website with more information on physical advertisements...the idea of a hotline is fantastic.

White gay man aged 18 years

They also suggested that such resources should be comprehensive enough so that adolescents do not have to hunt for information elsewhere:

The website should include all possible information about PrEP so that it’s a one stop shop and so there is no way to walk into it blind...it will inform and persuade because they can just look at it and know, rather than having...to look all of it up on their own.

White bisexual woman aged 19 years

### Example Campaigns From Focus Group Participants

At the end of the focus group, participants were given the following prompt: “We just asked you a bunch of questions about what a PrEP campaign for LGBTQ+ teens might look like. But some folks might prefer to share their thoughts visually.”

Participants were given the option to share graphics or existing sexual health content that evoked what they wanted from a PrEP campaign or to design an example from scratch. In this section, we provide several representative examples and their rationales.

**Figure 1:**
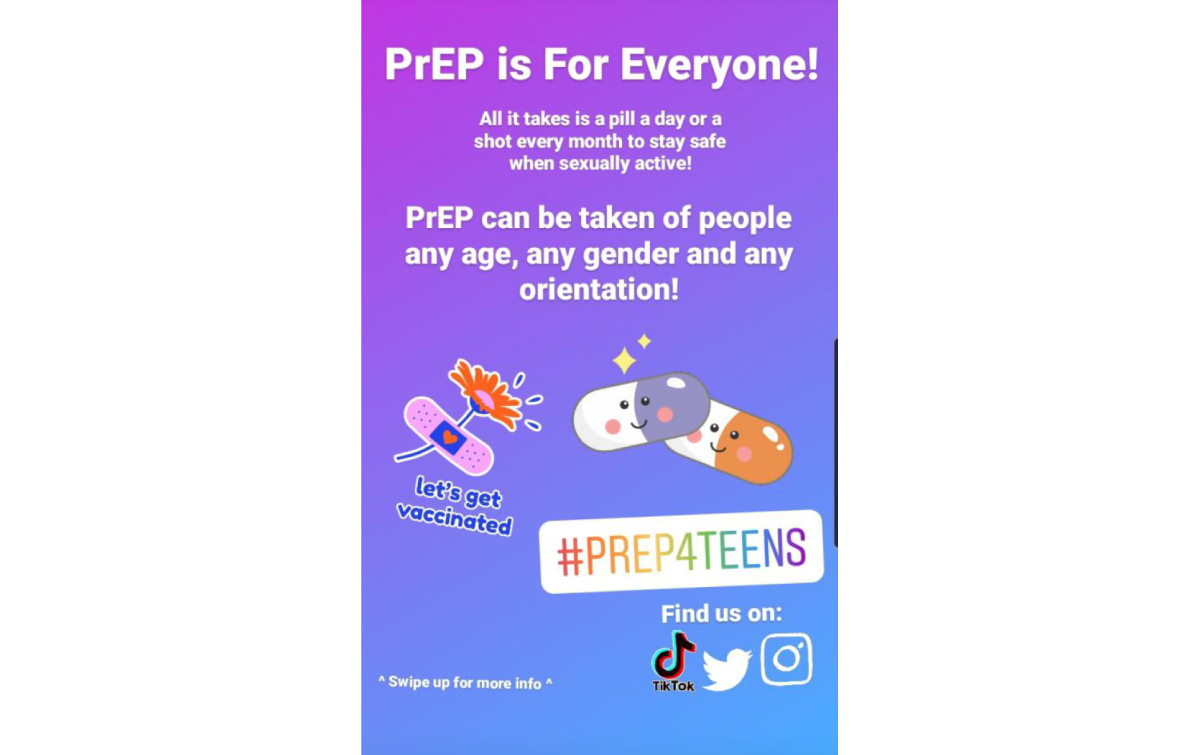
Social media pre-exposure prophylaxis (PrEP) campaign asset reflecting inclusive messaging and calls to action.

Participants who submitted an original entry most frequently gave examples formatted for social media, such as Instagram or TikTok. This participant described clarity, directness, and inclusion in their design:

The idea behind this one [Figure 1] was an Instagram ad that has simplicity, there’s a hook at the top, only 2 things to read, cute stickers, an option to swipe up for more info. And the two things I found most important were the hashtag that I was able to make rainbow! And the symbols for the other social media. I was able to get my point across that prep is for everyone, and a neat little reference is that the background somewhat resembles the bisexual flag, hinting that it doesn’t matter if you have sex with men or women [or both or anywhere in between] and that prep is still for you.

Hispanic gay man aged 17 years

**Figure 2:**
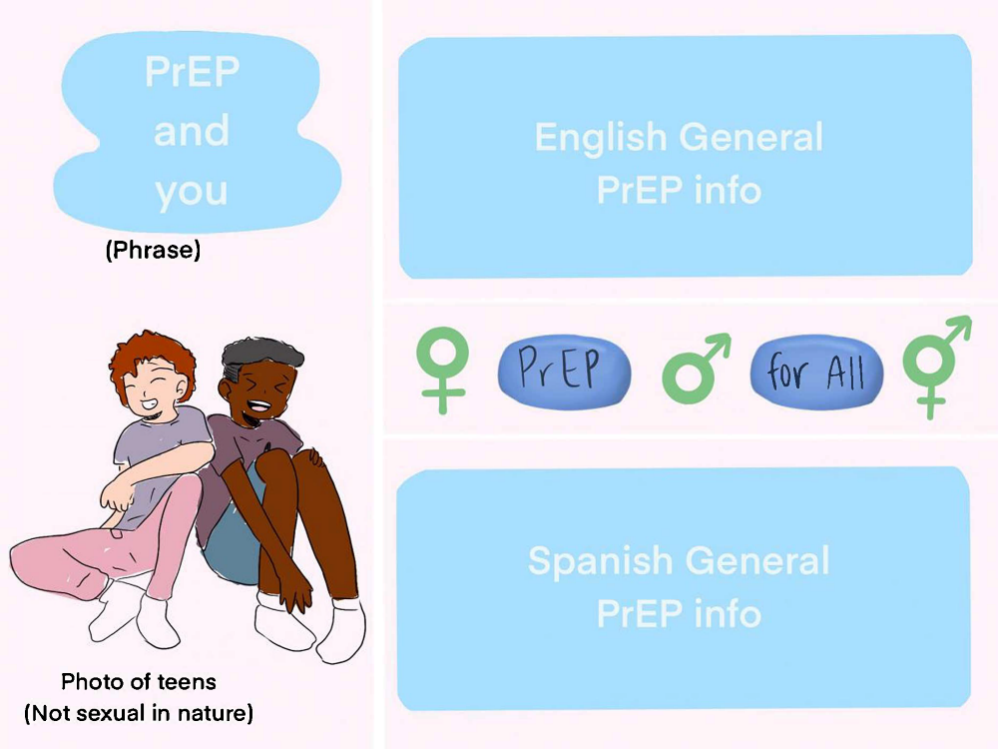
Social media pre-exposure prophylaxis (PrEP) campaign asset reflecting inclusive messaging and English- and Spanish-language information.

Others submitted examples that included information in both English and Spanish, referencing the conversation earlier in the focus group about the inclusion of Spanish speakers (Figure 2):

I figured that incorporating teens in the campaign in a nonsexual nature would...draw attention—these teens are also different, appearance-wise. For the information, I saw on a different forum that there should probably be information in both Spanish and English. Separating the information are gender symbols and PrEP pills that say, “PrEP for all” on them.

Black gay woman aged 18 years

One participant created a script for a commercial reminiscent of the US Trojan Man condom commercials [52] of the mid to late 1990s (Textbox 2):

I wrote a quick script for a hypothetical commercial. It’s aimed at a [younger?] teen audience and ideal for platforms like TikTok, Snapchat, and Instagram. I think it could be either live-action or animated. This one would maybe be best aimed at teens who have not yet started having sex considering that’s the situation in the commercial. The characters can be any gender/body type/sexual orientation. I also wrote it to be slightly raunchy since that will certainly appeal to/get teens attention.

White bisexual man aged 19 years

**Excerpt of proposed commercial or brief video for social marketing campaign.**

INT. A TEEN BOY'S ROOM LATE AT NIGHT 

Two teens make out in bed until TEEN 1 pulls away. 

TEEN 1 

You sure you're ready? 

TEEN 2 

(smiling warmly) 

Mhm. 

TEEN 2 leans into continue kissing TEEN 1 when CAPTAIN PrEP, a caped, over-the-top superhero with an anti-HIV logo or a pill logo on their shield, flies through the wall. The terrified teens scream. 

CAPTAIN PrEP 

Fear not, young horndogs. It is only Captain PrEP here to make sure you don't get HIV. 

TEEN 1 

What the f*ck, you destroyed my room! 

Captain PrEP is unfazed.

CAPTAIN PrEP 

(earnestly) 

We all make sacrifices. But your sexual health shouldn't be one of them. 

## Discussion

### Principal Findings

PrEP has the potential to dramatically reduce the number of HIV infections among adolescents in the United States. However, its use among young people remains low, with individual studies reporting that <0.5% of adolescents who may benefit from PrEP have used it [53,54]. Social marketing campaigns have the potential to improve PrEP awareness and uptake [24,26,32]; however, limited research has explored what adolescents want out of adolescent-centered PrEP campaigns. To our knowledge, this is among the first studies on SGM adolescents’ perspectives on existing and future PrEP marketing and implementation. Our findings, framed around the 4 Ps of social marketing, suggest that previous sexual health campaigns tailored for SGM adults, or adolescents in general, do not meet the needs of SGM adolescents and that youths themselves can be involved in conceptualizing the content and implementation of PrEP campaigns [43]. In this section, we discuss concrete takeaways from the focus groups that public health practitioners and implementers may consider incorporating into adolescent PrEP campaigns.

Overall, what adolescents wanted from PrEP campaigns aligned with our findings from interviews with adults who worked with or parented LGBTQ+ adolescents about what should go into an adolescent-centered PrEP campaign [24]. For example, adults wanted adolescent-centered PrEP campaigns to offer basic information about PrEP (eg, safety, how it works, access, and costs) that was inclusive, destigmatizing, and aligned PrEP with adolescents’ priorities (eg, romantic relationships). In addition, adults wanted concrete, actionable information for adolescents to act on (eg, call this hotline and go to this clinic), delivered through social media and physical spaces where adolescents spend most of their time. For instance, regarding *product* and *price*, SGM adolescents expressed a desire for basic PrEP information in these campaigns that address potential barriers and costs adolescents may encounter. Several years after PrEP’s Food and Drug Administration approval for adolescents, access to consumer-facing, adolescent-specific information on PrEP in the United States remains sparse. Although there are some examples of consumer-facing information that indicates that adolescents can take PrEP [55], there are few details addressing the developmental issues that surfaced in this study and previous research on adolescents and PrEP [8,11,12]. An adolescent-focused social marketing campaign could offer answers to the following questions, some of which may need to be tailored to reflect current state laws and policies: Do I need to involve my parents in the decision to take PrEP, because in some states, PrEP is explicitly included in mature minors’ right to health care, and in others, it is unclear or implied [56]? What health care providers or clinics can prescribe PrEP to minors, as web-based PrEP locator tools lack information about whether locations serve adolescents? I have never been to a health care provider on my own, so how do I prepare for a PrEP appointment? How do I pay for PrEP without my parents knowing?

Moreover, because PrEP requires consistent health care engagement (eg, for oral PrEP, quarterly appointments are recommended for HIV testing and lab work to check for side effects), campaigns should offer resources that help youth feel more comfortable independently accessing care given that they may have limited experience doing so. Options to cover or defray PrEP medication and related follow-up care should be made explicit (eg, coupons from pharmaceutical companies and requirements for insurance companies to cover PrEP prescriptions and services via the United States Affordable Care Act).

In general, participants in this study were more concerned about how to access PrEP than its side effects, suggesting that apart from basic information on PrEP to increase its awareness, campaigns should focus on demystifying the process of obtaining, paying for, and sustaining PrEP for adolescents. Highlighting real adolescents’ experiences pursuing PrEP can make a social campaign more relatable, according to our results. In a different qualitative study by our team, 100 adolescents who had successfully accessed PrEP described different strategies, barriers, and facilitators they encountered as well as their experiences of using PrEP [57]. Sharing anecdotes from these youths and others could be incorporated into a future PrEP campaign to ground the messages in reality [57]. Regarding *place* and *promotion*, participants described wanting a primarily digital campaign implemented via social media. SGM adolescents are heavy users of social media [58-60], where they often seek and consume health content, and consequently, a digital-first campaign could reduce adolescents’ embarrassment and stigma (which was relatively high in this sample) by reaching them where they already are. Moreover, additional suggestions indicated that participants wanted a campaign that acted as a “one-stop shop,” short of visiting a physical clinic. In other words, adolescents’ responses suggested that they wanted a campaign that not only raised awareness and offered education, but also included resources that could facilitate their ability to ask questions relatively anonymously and on demand (eg, a text line) as well as connections to PrEP care (eg, telehealth PrEP consultations). Nonetheless, adolescents also identified potential in-person spaces that could promote a PrEP campaign or connect adolescents to PrEP services, such as gender-sexuality alliances, offices of family medicine physicians or pediatricians, or public libraries. However, given rapidly increasing legislation proposing to restrict sexual, reproductive, gender-affirming, and LGBTQ+-inclusive care across the United States [61,62], one must consider the local social and cultural climate and policies to determine whether it is feasible, acceptable, and safe to implement campaign materials promoting adolescent sexual health services.

One important consideration for promotion is that at the time this study was conducted, self-service social media advertising platforms (eg, Meta, which includes Facebook and Instagram) permitted users to target advertisements to minors based on their demographics, such as gender, presumed sexual orientation identity, and race or ethnicity. However, since early 2023, in the United States, significant restrictions have been placed on social media advertising to minors that would preclude the ability to do market segmentation, in other words, focus educational campaigns on young populations who need it most. Thus, public health professionals wanting to reach minors via digital spaces may need to share their campaigns more broadly with adolescents and hope that LGBTQ+-inclusive messaging and imaging will reach their intended audiences. Alternatively, they will need to identify different means of sharing health campaign materials that do not solely rely on social media advertising, which is also increasingly more expensive. These means could include reaching youth-serving organizations with a digital presence instead of attempting to reach individual adolescents themselves or returning to a focus on in-person events where adolescents are present.

Regarding how content is displayed, participants advised against including sexualized images that are common in adult-focused sexual health campaigns. Instead, participants suggested bright, youthful imagery with concise and affirming messaging delivered by trusted messengers (eg, supportive parental figures, adolescents, and near peers). Results also indicated the need to strike a balance between purposefully inclusive, “PrEP is for all” campaign messaging and information intended to reach populations in demographic groups at higher HIV transmission risk or those with unique medical or health care access considerations, such as transgender, nonbinary, and rural or suburban youth. This preference stands in contrast to PrEP awareness campaigns that tend to adopt a targeted approach to specific populations at greatest risk of HIV transmission. Consistent with literature showing that adolescents value diversity in media [63], participants showed a strong desire for a PrEP campaign that emphasizes diversity across multiple dimensions. However, without some market segmentation that considers local context and needs regarding PrEP, a campaign can run the risk of seeming too generic and impersonal and, consequently, may not reach those who need the resources the most. Moreover, participants repeatedly expressed a distaste for marketing materials that appeared too “corporate” and polished, which suggested that community members who were young, LGBTQ+, and Black, Indigenous, and people of color were not as involved in campaign design. This speaks to the need for adolescent HIV prevention campaign designers to involve community members who are the intended recipients throughout the process, which can also increase trust and buy-in when the campaign is implemented.

Finally, to better understand what participants envisioned adolescent-centered PrEP campaign materials might look like, we included a participatory design exercise at the end of the focus group. Such approaches have been used previously in adolescent sexual health intervention design and have been suggested to improve both youth well-being and youth-centered programming [64,65]. This exercise enabled us to begin to understand how participants’ text responses in the focus group data manifested in their design choices and priorities. Ultimately, these youth-generated materials can be used to inspire the direction of PrEP campaign components, ensuring that design choices are not guided solely by researchers’ interpretations of focus group data but by adolescents’ own visions as well. In fact, these findings, in part, formed the basis of our own adolescent-centered PrEP social marketing campaign, PrEP4Teens [66], launched in late 2023, the development of which will be described in a future publication.

### Limitations

While this study has valuable insights, it is important to acknowledge its limitations. The participants were predominantly adolescents who had heard of PrEP before, with a larger proportion of transgender and nonbinary teenagers as well as cisgender sexual minority girls, compared to what is typically seen in HIV prevention research focusing on those at the highest behavioral risk for HIV acquisition (eg, sexually active sexual minority male individuals and transfeminine individuals who engage in unprotected anal sex). It is possible that adolescents who have not heard of PrEP before might want different information or have different concerns than those who are already somewhat familiar with PrEP. Moreover, those in groups at the highest risk for acquiring HIV may have different perspectives on the campaigns. However, arguably, as nationwide clinical guidelines suggest that all sexually active adolescents and adults should be informed about PrEP regardless of sexuality or gender [67], this sample composition can also be seen as a strength. As this study took place in the Chicago area, which is a large urban area with a historically progressive social and political climate, findings may not generalize to other environments. Furthermore, as this study was conducted in February to May 2021, when most in-person social behavioral research studies had not yet resumed during the COVID-19 pandemic, it is possible that our use of asynchronous web-based focus groups may not have been able to capture certain nuances that live, in-person focus groups would have (eg, body language and tone of voice).

### Conclusions

Developmentally tailored, SGM adolescent–inclusive information about PrEP and where to access PrEP services is critical to contribute to US efforts to end the domestic HIV epidemic. Social marketing campaigns that directly reach adolescents have great potential to address these needs quickly and equip them with the tools to make informed decisions about their sexual health. Public health professionals can consider developing their own adolescent-centered PrEP campaigns guided by our findings and make their campaigns responsive to local needs in partnership with local adolescents and adult stakeholders who support or parent adolescents. Public health dissemination and implementation researchers should consider ways to measure and evaluate the reach and impact of PrEP campaigns on adolescents’ PrEP awareness, interest, and uptake—perhaps, in part, by adding questions about social marketing campaign exposure and PrEP uptake to large public health surveys of adolescents (eg, Youth Risk Behavior Surveillance Survey by Centers for Disease Control and Prevention [68])—and explore how such patterns may differ regionally and by social-political climate. Having said that, as parents, health care providers, and other supportive adults are also critical gatekeepers of adolescents’ access to health care and health information, additional research should examine and design educational PrEP campaigns for these groups.

## Abbreviations

LGBTQ+: lesbian, gay, bisexual, transgender, and queer
PrEP: pre-exposure prophylaxis
SGM: sexual and gender minority
STI: sexually transmitted infection

## Data Availability

The datasets generated during and analyzed during this study are not publicly available as they contain identifiable information about a socially marginalized group of minor adolescents, but deidentified data are available from the corresponding author on reasonable request.
